# Is *Lactobacillus* spp. beneficial in human semen? Systematic review

**DOI:** 10.5935/1518-0557.20250023

**Published:** 2025

**Authors:** Jenniffer Puerta-Suárez, Walter D. Cardona Maya

**Affiliations:** 1 Reproduction Group, Department of Obstetrics and Gynecology, Medical School, University of Antioquia - UdeA, Antioquia, Colombia; 2 Reproduction Group, Department of Microbiology and Parasitology, Medical School, University of Antioquia - UdeA, Antioquia, Colombia

**Keywords:** *Lactobacillus* spp, semen, fertility, bacteria, spermatozoa

## Abstract

The present systematic review aimed to evaluate the effects of *Lactobacillus* spp. on human semen. The review was conducted until May 2023, according to the PRISMA Statement and using Scopus and PubMed databases. The protocol was registered on PROSPERO (CRD42024519245). All published original investigation peer-reviewed articles in English and Spanish related to *Lactobacillus* spp. and human semen parameters were eligible. The quality assessment used The Joanna Briggs Institute (JBI) Critical Appraisal Checklist and QUIN tool. In total, 35 articles were included. *Lactobacillus* spp. is frequently detected in semen, and its impact on seminal quality is controversial, especially regarding the methodology used. The intake of probiotics with *Lactobacillus* spp. can improve semen parameters. In conclusion, *Lactobacillus* spp. appears to have a beneficial role in semen quality. Probiotics could have a good impact on improving semen quality; however, further studies are required.

## INTRODUCTION

Infertility is an increasing condition, where the male factor alone or in combination with the female factor is responsible for 40 to 50% of cases (Weng *et al.*, 2014)only few types of bacteria were taken into consideration while using PCR-based or culturing methods. Here we present an analysis approach using next-generation sequencing technology and bioinformatics analysis to investigate the associations between bacterial communities and semen quality. Ninety-six semen samples collected were examined for bacterial communities, measuring seven clinical criteria for semen quality (semen volume, sperm concentration, motility, Kruger’s strict morphology, antisperm antibody (IgA. Causes of infertility include infections of the male genitourinary tract in approximately 15% of cases (Mändar *et al.*, 2015)current knowledge of the male microbiome is scarce, and parallel studies examining couples are extremely rare. In this work, we aimed to compare seminal and vaginal microbiomes in couples and to assess the influence of sexual intercourse on vaginal microbiome. The study included 23 couples. Microbiomes of semen and vaginal fluid (preand post-intercourse. Infection and inflammation of the different organs that make up the male reproductive tract, such as the prostate, seminal vesicles, vas deferens and epididymis, are frequent reasons for andrology consultation (Grande *et al.*, 2022)commonly treated with antibiotics alone. However, in approximately 40% to 50% of patients, persistent infection is detected. Intestinal dysbiosis is involved in the pathogenesis of prostatitis. We aimed to evaluate the efficacy of antibiotic treatment in association with a specific probiotic supplementation. A total of 104 infertile patients, with microbiological analysis on semen and/or prostatic secretions positive for Gram-negative bacteria, have been enrolled. All patients received antibiotic treatment with fluoroquinolones. In total, 84 patients received a commercial association of Enterococcus faecium and Saccharomyces boulardii during antibiotic treatment, followed by treatment with Lactobacilli. After the treatment, a complete microbiological analysis was repeated. Polymicrobial infections have been observed in 11% of patients, while infections due to a single germ were reported in 89% of the patients. After the treatment was performed, a complete eradication with negative semen culture and microbiological analysis on prostatic secretion was observed in 64 of 84 patients (76.2%. However, microorganisms in the male reproductive tract are not always associated with disease. Recent studies have evaluated the presence of the microbiota in different portions of the male reproductive tract and its impact on seminal quality. Among the microbial species that belong to the urogenital microbiota is *Lactobacillus* spp., which, although its function in the balance of the vaginal microbiome has been widely elucidated, its role in the male reproductive tract is still unclear (Weng *et al.*, 2014; Puerta-Suárez & Cardona Maya, 2024)only few types of bacteria were taken into consideration while using PCR-based or culturing methods. Here we present an analysis approach using next-generation sequencing technology and bioinformatics analysis to investigate the associations between bacterial communities and semen quality. Ninety-six semen samples collected were examined for bacterial communities, measuring seven clinical criteria for semen quality (semen volume, sperm concentration, motility, Kruger’s strict morphology, antisperm antibody (IgA.

*Lactobacillus* is a bacterial genus composed of multiple species whose main characteristic is that they are considered benign colonizers of multiple anatomical sites and even other animals. This genus especially predominates in the gastrointestinal tract, and the urinary and genital tracts, are the primary colonizers of the vagina. At an industrial level, they are used to produce fermented dairy foods. The main characteristic of Lactobacillus is the ability to produce lactic acid, which creates an acidic environment that limits the growth of other pathogenic microorganisms such as anaerobic bacteria, for example, *Peptostreptococcus anaerobius* and *Prevotella bivia* (Grande *et al.*, 2022)commonly treated with antibiotics alone. However, in approximately 40% to 50% of patients, persistent infection is detected. Intestinal dysbiosis is involved in the pathogenesis of prostatitis. We aimed to evaluate the efficacy of antibiotic treatment in association with a specific probiotic supplementation. A total of 104 infertile patients, with microbiological analysis on semen and/or prostatic secretions positive for Gram-negative bacteria, have been enrolled. All patients received antibiotic treatment with fluoroquinolones. In total, 84 patients received a commercial association of Enterococcus faecium and Saccharomyces boulardii during antibiotic treatment, followed by treatment with Lactobacilli. After the treatment, a complete microbiological analysis was repeated. Polymicrobial infections have been observed in 11% of patients, while infections due to a single germ were reported in 89% of the patients. After the treatment was performed, a complete eradication with negative semen culture and microbiological analysis on prostatic secretion was observed in 64 of 84 patients (76.2%.

The initiation of sexual intercourse promotes a change in the male and female genitourinary microbiota, which increases the dissemination of microorganisms responsible for sexually transmitted infections but also exerts changes in the genitourinary microenvironments that can be associated with health or disease. However, studies that evaluate the microbiota of couples are relatively few and present methodological problems associated with defining bacterial species associated with health or disease (Mändar *et al.*, 2015)current knowledge of the male microbiome is scarce, and parallel studies examining couples are extremely rare. In this work, we aimed to compare seminal and vaginal microbiomes in couples and to assess the influence of sexual intercourse on vaginal microbiome. The study included 23 couples. Microbiomes of semen and vaginal fluid (preand post-intercourse. Microorganisms can interact with the spermatozoa during their transport, affecting their quality. Therefore, it is essential to consider the effect of *Lactobacillus* on the male reproductive tract and its impact on seminal quality. This systematic review aims to evaluate the effects of *Lactobacillus* spp. on human semen.

## MATERIALS AND METHODS

The systematic literature search followed the Preferred Items for Systematic Reviews and Meta-analysis (PRISMA) reporting guidelines (Page *et al.*, 2021). The following search string was used for PubMed and Scopus: *Lactobacillus* spp. AND human AND (semen or sperm).

### Eligibility criteria

The following PICO (population, intervention, comparator, and outcome) elements were set as inclusion criteria: *i*) population: male patients; *ii*) intervention: assessment of *Lactobacillus* spp. in human semen; *iii*) comparison: self-comparison or independent controls (placebo or no treatment); and *iv*) outcome: change in the semen parameters. We will include all published original investigation peer-reviewed articles in English and Spanish related to *Lactobacillus* spp. and human semen parameters until May 2023. All duplicates, publications related to animal models and reviews were excluded.

### Data extraction and quality assessment

**Figure 1:**
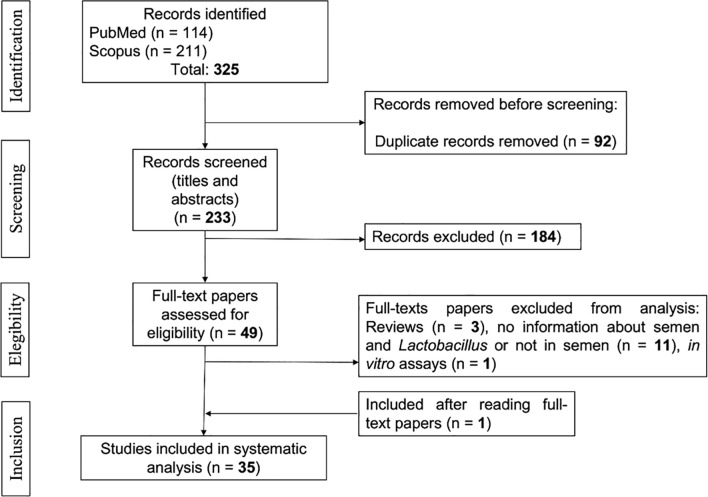
Flow diagram of the study selection process.

Both authors independently reviewed the titles and abstracts of the identified studies to assess their eligibility based on predefined inclusion and exclusion criteria. They then retrieved full-text articles of potentially relevant studies for further evaluation. Relevant information from the selected studies using a standardized data extraction form during the data collection. This form captured the study’s main characteristics, title, outcome measures, and other relevant information. The data extraction was conducted meticulously to ensure accurate and consistent data collection (Figure 1). Any reviewers’ disagreements were resolved through discussion to ensure a consensus-based selection process. The study protocol was registered with the Prospero International Prospective Register of Systematic Reviews (CRD42024519245).

Finally, methodological quality and potential risk of bias were assessed using The Joanna Briggs Institute (JBI) Critical Appraisal Checklist and QUIN tool were used to assess the potential risk of bias (Supplementary Table S1).

## RESULTS

After applying the inclusion and exclusion criteria to the articles, the researchers independently selected 35 articles. Additionally, with the manual review of the manuscripts, another article was considered among the researchers to be included. Considering the diversity of methodologies used to evaluate the effects of *Lactobacillus* spp. on semen quality, the manuscripts were classified according to four methodologies: *a*) the detection of the presence of *Lactobacillus* spp. in semen samples; *b*) the effect of the intake of prebiotics rich in *Lactobacillus* spp. on semen quality; *c*) the *in vitro* effect of incubating *Lactobacillus* spp. on sperm physiology, and *d*) detecting different *Lactobacillus* spp. in semen samples from men and their associations with different alterations, diseases or conditions (Table 1).

**Table 1:** Main findings concerning the effects of *Lactobacillus* spp. on semen quality.

Autor (reference)	Country	Population and results
**Detection of the presence of *Lactobacillus* spp. in semen samples**		
Eggert-Kruse *et al.* (1992)	Germany	1000 infertile couples. *Lactobacillus* spp. was detected in 56.8% (531 of 935) of the cervix samples and 13.5% (117 of 869) of the semen samples.
Voroshilina *et al.* (2020)	Russia	634 semen samples. *Lactobacillus* spp. was detected in 125 (19.7%) semen samples.
Voroshilina *et al.* (2021)	Russia	227 normooospermia semen samples. *Lactobacillus* spp. is detected in 26 (11.5%) semen samples. In half of the semen samples classified as normozoospermic, the presence of obligate anaerobes and *Lactobacillus* spp. was detected.
Rivera *et al.* (2022)	Colombia	81 asymptomatic men for urogenital infections. The most frequent microorganism found in semen was *Lactobacillus* spp. (70%). No association was found between *Lactobacillus* spp. detection and seminal quality.
**Intake of prebiotics rich in *Lactobacillus* spp.**		
Valcarce *et al.* (2017)	Spain	9 asthenozoospermic men. Sperm motility was improved after the treatment, sperm DNA fragmentation and intracellular H_2_O_2_ level were reduced after probiotic administration.
Maretti & Cavallini (2017)	Italy	41 infertile men were divided into consumed prebiotic (Flortec) and control (food starch). The semen parameters (volume, concentration, motility, and sperm morphology) and levels of follicle-stimulating hormone (FSH), luteinizing hormone (LH), and testosterone significantly improved after intake of prebiotic.
Helli *et al.* (2022)	Iran	52 men with idiopathic oligoasthenoteratozoospermia. Daily supplementation with 500mg of probiotics for 10 weeks significantly improves semen parameters: volume, total sperm count, sperm concentration, sperm total motility and vitality.
Abbasi *et al.* (2021)	Iran	56 infertile men were divided in control group (n=28) and treatment group (n=28). In men who consumed Familact, an increase in sperm quality (concentration, motility, morphology, lipoperoxidation and sperm DNA fragmentation) was observed.
Grande *et al.* (2022)	Italy	104 men with primary infertility. A complete eradication with negative semen culture and microbiological analysis on prostatic secretion was observed in 64 of 84 (76.2%) patients receiving probiotics and antibiotics, while only 10 of 20 (50%) patients receiving antibiotics alone.
Asadi *et al.* (2023)	Iran	78 men candidates for subinguinal microscopic varicocelectomy. At 3 months after intake of prebiotic, sperm concentration, normal morphology, semen volume and motile sperm were better than the placebo group.
***in vitro* effect of the incubation of *Lactobacillus* spp. species on sperm physiology**		
Barbonetti *et al.* (2011)	Italy	10 normozoospermic men who consented for fertility problems. *Lactobacillus* spp. prevented sperm lipid peroxidation induced *in vitro* by a ferrous ion promoter, which preserves sperm motility and viability.
Barbonetti *et al.* (2013)	Italy	6 normozoospermic healthy donors. Soluble factors of *Lactobacillus* spp. prevented membrane lipid peroxidation of *E. coli*-exposed spermatozoa, thus preserving their motility. Mitochondrial effects of *E. coli* are not prevented by the mix of *Lactobacillus* spp. *Lactobacillus* spp. could protect spermatozoa in the presence of vaginal disorders by preventing ROS-induced membrane damage.
Raad *et al.* (2023)	Lebanon	30 infertile men. *Lactobacillus plantarum* secretions (10^8^ CFU per mL) protected sperm DNA integrity and motility compared to the freezing medium without *Lactobacillus plantarum* secretions.
**Detection of different Lactobacillus spp. species in semen samples from men and their association with different alterations, diseases or conditions**		
Hillier *et al.* (1990)	USA	37 men from infertility clinic. An average of 5.3 microorganisms per ejaculate was found, 16% of the samples had *Lactobacillus*.
Kiessling *et al.* (2008)	USA	29 Men undergoing fertility evaluation and 5 men with vasectomy. *Lactobacillus* spp. bacterial DNA was detected in 10 semen samples.
Ivanov *et al.* (2009)	Russia	108 men: 48 healthy men and 60 men with chronic prostatitis syndrome. *Lactobacillus* spp. detection in healthy men was 28 (58.3%, 3.5±0.5 mean bacterial count log 10 CFU/mL), while in patients with prostatitis, it was 20 (33.3% of men) with 3.1±0.2 mean bacterial count log 10 CFU/mL.
Weng *et al.* (2014)	Taiwan	96 men with primary infertility. The most abundant species of bacteria in semen are *Lactobacillus iners* (14.09%). *Lactobacillus crispatus* was not only associated with sperm elongation and with Kruger’s strict morphology.
Chen *et al.* (2018)	China	The fertile control (n=5) group, the obstructive azoospermia (n=6) group and the non-obstructive azoospermia (n=6) group. 398 common operational taxonomic units were identified, of which 27 belonged to *Lactobacillus* spp. (6.79% in the fertile group), whereas it accounted for 17.98% and 17.24% in the obstructive and no obstructive azoospermia groups, respectively.
Mändar *et al.* (2017)	Estonia	67 men: 21 with chronic prostatitis and 46 controls. The most remarkable difference between the groups appeared in the counts of *Lactobacillus* spp. that were higher in healthy men than prostatitis patients (median 27% *vs.* 20.2%, p=0.05), especially on behalf of *Lactobacillus iners* (14.2% *vs.* 9.8%, p=0.013).
Monteiro *et al.* (2018)	Portugal	89 cases and 29 controls. *Lactobacillus* spp. was found at very low abundances in all pooling samples, with the highest proportion registered in pool control (0.6%) and the lowest in pool hyperviscosity (>0.1%).
Amato *et al.* (2020)	Italy	23 couples. Relative abundance of *Lactobacillus* spp. was performed for the seminal microbiome, revealing differences in the relative abundance of *Lactobacillus* spp. among the cohort of idiopathic infertile men.
Baud *et al.* (2019)	Switzerland	26 men with normal semen analysis and 68 men with at least one anormal sperm parameter. The relative abundance of *Lactobacillus* spp. was greater in samples with normal sperm morphology.
Yang *et al.* (2020)	China	159 study participants: 22 patients with oligoasthenospermia, 58 patients with asthenospermia, 8 patients with azoospermia, 13 patients with oligospermia, and 58 healthy controls. *Lactobacillus* spp. was among the most abundant genera commonly found in seminal plasma.
Štšepetova *et al.* (2020)	Estonia	50 infertile men. The most abundant genera of bacteria in the semen before washing (73.3%), and IVF culture solution (35.5%) was *Lactobacillus* spp.
Motamedifar *et al.* (2020)	Iran	350 men: 200 infertile and 150 fertile men. The prevalence of bacteriospermia in the semen of the infertile group was significantly higher than that in the fertile group (48% *vs.* 26.7%, *p*<0.001). Moreover, bacteriospermia among the infertile group was associated with higher abnormality in concentration, motility, and sperm morphology (*p*<0.001). In the control group, *Lactobacillus* spp. (17.3%) was the most isolated bacteria.
Pochernikov *et al.* (2020)	Russia	210 men in two groups, Control group: 105 men without *Lactobacillus* spp. in the ejaculate and Treatment group 105 men with the presence of *Lactobacillus* spp. in semen. *Lactobacillus* spp. is associated with oligoasthenoteratozoospermia (*p*<0.01), decreased sperm concentration (*p*=0.01), decreased sperm motility (*p*<0.01) and morphological abnormalities (*p*<0.01).
Okwelogu *et al.* (2021)	Nigeria	36 infertile couples. *Lactobacillus* spp. Was more abundant bacteria in semen and vaginal swabs. The family taxa Lactobacillaceae and the *Lactobacillus* genus were significantly higher in the vaginal swabs than in the semen (61.7 *vs.* 43.9%). The semen samples of men with positive IVF clinical outcomes were significantly colonized by the *Lactobacillus jensenii*.
Yao *et al.* (2022)	China	87 seminal samples: 33 with a normal seminal leukocyte count and 54 samples with leukocytospermia, 48.1% of men with leukocytospermia had *Lactobacillus* spp.
Garcia-Segura *et al.* (2022)	Spain	56 participants: 14 controls healthy normozoospermic semen donors with no infertility diagnosis and 42 idiopathic normozoospermic infertile patients. seminal microbiota was mainly composed of four phyla: Firmicutes (59%), Proteobacteria (19%), Actinobacteria (8%), and Bacteriodetes (5%).
Puerta Suárez *et al.* (2022)	Colombia	10 chronic prostatitis-like syndrome and 11 fertile donors. The control group had more *Lactobacillus* spp in the semen (58%) compared to the prostatitis group (20%).
Koort *et al.* (2023)	Estonia	Couples with assisted reproductive technology procedures (n=97) and fertile couples (n=12). The prevalence of *Lactobacillus* spp. was lower in semen samples from assisted reproduction techniques men.
Cao *et al.* (2023)	China	53 men: 12 controls with normal semen parameters, 12 asthenozoospermia, 6 with oligozoospermia, 9 with several oligozoospermia or azoospermia, and 14 with hyperviscosity. *Lactobacillus* spp. is positively correlated with sperm concentration and total sperm count.
Saldarriaga López *et al.* (2024)	Colombia	22 samples from men with symptoms of chronic prostatitis and 31 asymptomatic men (control group). The volunteers from the group of men with symptoms of chronic prostatitis presented less frequently the DNA of *A. vaginae* (4.4% *vs*. control group 9.7%), *G. vaginalis* (45.5% *vs.* control group 54.8%), *L. crispatus* (9.1% *vs.* control group 16.1%) and *L. iners* (45.5% *vs.* control group 58.1%).
Osadchiy *et al.* (2024)	USA	73 men: 42 with normal semen analysis and 31 with anormal semen analysis. Participants with normal sperm motility showed a lower abundance of *Lactobacillus iners* (*p*=0.0464) than those with abnormal sperm motility, mean proportion was 9.4% *vs*. 2.6%.
Vajpeyee *et al.* (2024)	India	69 men with normal semen parameters and 166 men with at least 1 normal parameter. The relative distribution of *Lactobacillus* spp. and *Prevotella* in the normal and abnormal semen groups were different. In the abnormal semen group, the incidence of *Lactobacillus* spp. probiotics was lower, and the frequency of *Prevotella* was higher.

## DISCUSSION

Traditionally, the presence of bacteria in seminal plasma has been considered an indicator of poor sperm quality. However, today, we know that not all species of bacteria can be associated with alterations in seminal quality. A clear example is *E. coli*, detected in 94 of 96 samples from infertile men, yet no associations with seminal quality can be identified (Weng *et al.*, 2014)only few types of bacteria were taken into consideration while using PCR-based or culturing methods. Here we present an analysis approach using next-generation sequencing technology and bioinformatics analysis to investigate the associations between bacterial communities and semen quality. Ninety-six semen samples collected were examined for bacterial communities, measuring seven clinical criteria for semen quality (semen volume, sperm concentration, motility, Kruger’s strict morphology, antisperm antibody (IgA. Additionally, the management of male urogenital tract infections, especially infections associated with the prostate, is treated with fluoroquinolones, given the penetration and bioavailability of this antibiotic (Grande *et al.*, 2022)commonly treated with antibiotics alone. However, in approximately 40% to 50% of patients, persistent infection is detected. Intestinal dysbiosis is involved in the pathogenesis of prostatitis. We aimed to evaluate the efficacy of antibiotic treatment in association with a specific probiotic supplementation. A total of 104 infertile patients, with microbiological analysis on semen and/or prostatic secretions positive for Gram-negative bacteria, have been enrolled. All patients received antibiotic treatment with fluoroquinolones. In total, 84 patients received a commercial association of Enterococcus faecium and Saccharomyces boulardii during antibiotic treatment, followed by treatment with Lactobacilli. After the treatment, a complete microbiological analysis was repeated. Polymicrobial infections have been observed in 11% of patients, while infections due to a single germ were reported in 89% of the patients. After the treatment was performed, a complete eradication with negative semen culture and microbiological analysis on prostatic secretion was observed in 64 of 84 patients (76.2%. However, we do not know the impact of this treatment on the disease or how it can affect species of bacteria that appear beneficial in the male genitourinary tract, as has been described for the female genital tract, especially the *Lactobacillus* family. Traditionally, *Lactobacillus* detection has become much more relevant in the vagina. This microorganism is associated with a healthy microenvironment, so its microscopic observation and semi-quantification of its presence become very important in disorders such as bacterial vaginosis. Also, cultures for the identification of Lactobacillus in semen samples may have limited value in routine seminal analysis since, like what happens with vaginal discharge samples, growth of different species of *Lactobacillus* can be obtained even in individuals with alterations in the microbiota, as occurs with bacterial vaginosis, which makes this test less valuable. However, molecular biology techniques have made it possible to determine the presence of this bacteria in other tracts, such as the male genitourinary tract. This detection can be done using the polymerase chain reaction (PCR). However, this methodology only allows the detection of a single species with each set of primers and results in presence or absence (Rivera *et al.*, 2022)in semen samples from apparently healthy men and correlate their presence with seminal quality.\nMETHODS: Semen samples from 81 healthy volunteers were collected, and semen parameters were analyzed. DNA extraction was performed using the phenol-chloroform technique, and the microorganisms were detected by the amplification of specific primers using polymerase chain reaction.\nRESULTS: DNA from at least one of the microorganisms was detected in 78 samples. The most frequent microorganism found in semen were: Lactobacillus spp. (70%. Using more robust techniques such as sequencing, on the other hand, allows for identifying multiple species of the genus and comparing their relative abundance in multiple anatomical sites, with the disadvantage of their cost and the time required to perform the analyses (Puerta Suárez *et al.*, 2022).

Different species of *Lactobacillus* cover many of the body surfaces, including the oral cavity, the vaginal cavity, the male genitourinary tract, and the gastrointestinal tract; this genus is one of the most important representatives of the intestinal microbiota and has a substantial impact on reproduction and fertility since dysbiosis of the intestinal microbiota can increase the permeability of the blood-testis barrier and affect the serum levels of FSH, LH and testosterone (Cao *et al.*, 2023)leading to increased male infertility. This study analyzed the microbiota of the gut, semen, and urine in individuals with semen abnormalities to identify potential probiotics and pathogenic bacteria that affect semen parameters and help develop new methods for the diagnosis and treatment of patients with semen abnormalities.\nMETHODS: We recruited 12 individuals with normal semen parameters (control group. Therefore, we can infer that the intestinal microbiota influences sperm production and physiology (Puerta-Suárez & Cardona Maya, 2024).

Another essential point to consider is that semen is a means of spreading microorganisms, but in fact, sexual relationships are, in themselves, a factor that influences fertility. Microorganisms characterized as sexually transmitted infections can be transmitted (Rivera *et al.*, 2022)in semen samples from apparently healthy men and correlate their presence with seminal quality.\nMETHODS: Semen samples from 81 healthy volunteers were collected, and semen parameters were analyzed. DNA extraction was performed using the phenol-chloroform technique, and the microorganisms were detected by the amplification of specific primers using polymerase chain reaction.\nRESULTS: DNA from at least one of the microorganisms was detected in 78 samples. The most frequent microorganism found in semen were: Lactobacillus spp. (70%, which has a high impact on public health; however, it also presents the opportunity to obtain beneficial species that reduce the risk of suffering from disorders such as prostatitis (Puerta Suárez *et al.*, 2022; Puerta-Suárez & Cardona Maya, 2024; Saldarriaga López *et al.*, 2024)antioxidant capacity, and pro-inflammatory cytokines in semen and seminal plasma samples were also quantified. Finally, the expression of the ROR-γT, FoxP3, and T-bet genes in semen and the presence of DNA of microorganisms associated with prostatitis in urine and semen were evaluated.\nRESULTS: When compared with fertile donors, volunteers with chronic prostatitis-like symptoms reported erectile dysfunction (0% vs. 10%, p = 0.2825.

Although studies on the effects of *Lactobacillus* spp. are controversial and sometimes contradictory, these variations are possibly associated more with the specific species of *Lactobacillus* spp. evaluated than with the methodology used to evaluate the impact of bacteria on semen quality. Therefore, future studies need to focus not only on evaluating the effect of this species on fertility, whether it is administered or identified its presence in the male genitourinary tract, but also on the different species of this family to try to elucidate the real impact on male fertility. This effect should also be evaluated in couples, considering that fertility is a couple’s problem, and that intercourse modifies the microbial composition (Mändar *et al.*, 2015)current knowledge of the male microbiome is scarce, and parallel studies examining couples are extremely rare. In this work, we aimed to compare seminal and vaginal microbiomes in couples and to assess the influence of sexual intercourse on vaginal microbiome. The study included 23 couples. Microbiomes of semen and vaginal fluid (preand post-intercourse.

Regarding the consumption of probiotics that include species of the *Lactobacillus*, although we have gathered evidence through this review that this consumption can improve sperm DNA, reduce reactive oxygen species, modify hormonal levels to improve sperm production, and even improve sperm volume and concentration, viability and mobility (Maretti & Cavallini, 2017; Valcarce *et al.*, 2017; Abbasi *et al.*, 2021; Helli *et al.*, 2022; Asadi *et al.*, 2023)Bracco; one sachet contains: Lactobacillus paracasei B21060 5 × 109 cells + arabinogalctan 1243 mg + oligo-fructosaccharides 700 mg + l-glutamine 500 mg, it is still pertinent to carry out studies that can demonstrate the mechanism of action that the intake of these bacteria can have on seminal quality. However, it should be clarified that the consumption of probiotics is a strategy that can be considered harmless if it is suggested to increase the consumption of fermented dairy products rich in *Lactobacillus* and that the benefits of this intervention may outweigh the associated risks.

Finally, this is a good approximation to describe the effect of *Lactobacillus* on semen quality. Bacteria in the genitourinary tract have traditionally been considered synonymous with disease. However, we now know that this statement is not entirely true. *Lactobacillus* in the male genitourinary tract is associated, as in the gastrointestinal tract and the female genital tract, with a healthy microbial environment, limiting pathogenic bacteria. Among the limitations found when evaluating the impact of *Lactobacillus* on semen quality, we must highlight the differences in the methodologies proposed in the different articles. The microbiota and its impact on health is a new topic, in which very interesting findings are being made. However, more research is still needed to support, from cellular and molecular biology, the impact that *Lactobacillus* has on male sexual and reproductive health in general. However, it is hoped that this review will at least provide a state-of-theart of such a new topic that can modulate semen quality.

## CONCLUSION

*Lactobacillus* spp. is a frequent colonizer of the male urogenital tract and appears beneficial in semen quality, although controversial, especially regarding the methodology used, and discrepancies in the results may also be associated with specific *Lactobacillus* genus. On the other hand, probiotics could have a good impact on improving semen quality. Therefore, new studies are needed to evaluate the impact of different *Lactobacillus* species on seminal quality and fertility.

## Appendix

**Supplementary Table S1:** The Joanna Briggs Institute (JBI) Critical Appraisal Checklist and QUIN tool.

Reference		Q1	Q2	Q3	Q4	Q5	Q6	Q7	Q8						X/8	
Eggert-Kruse *et al.* (1992)	Germany	Yes	Yes	Yes	Yes	Yes	Yes	Yes	Yes						8/8	
Voroshilina *et al.* (2020)	Russia	Yes	Yes	Yes	Yes	Yes	Yes	Yes	Yes						8/8	
Voroshilina *et al.* (2021)	Russia	Yes	Yes	Yes	Yes	Yes	Yes	Yes	Yes						8/8	
Rivera *et al.* (2022)	Colombia	Yes	Yes	Yes	Yes	Yes	Yes	Yes	Yes						8/8	
Hillier *et al.* (1990)	USA	Yes	Yes	Yes	Yes	Yes	Yes	Yes	Yes						8/8	
Kiessling *et al.* (2008)	USA	Yes	Yes	Yes	Yes	Yes	Yes	Yes	Yes						8/8	
Ivanov *et al.* (2009)	Russia	Yes	Yes	Yes	Yes	Yes	Yes	Yes	Yes						8/8	
Weng *et al.* (2014)	Taiwan	Yes	Yes	Yes	Yes	Yes	Yes	Yes	Yes						8/8	
Chen *et al.* (2018)	China	Yes	Yes	Yes	Yes	Yes	Yes	Yes	Yes						8/8	
Mändar *et al.* (2017)	Estonia	Yes	Yes	Yes	Yes	Yes	Yes	Yes	Yes						8/8	
Monteiro *et al.* (2018)	Portugal	Yes	Yes	Yes	Yes	Yes	Yes	Yes	Yes						8/8	
Amato *et al.* (2020)	Italy	Yes	Yes	Yes	Yes	Yes	Yes	Yes	Yes						8/8	
Baud *et al.* (2019)	Switzerland	Yes	Yes	Yes	Yes	Yes	Yes	Yes	Yes						8/8	
Yang *et al.* (2020)	China	Yes	Yes	Yes	Yes	Yes	Yes	Yes	Yes						8/8	
Štšepetova *et al.* (2020)	Estonia	Yes	Yes	Yes	Yes	Yes	Yes	Yes	Yes						8/8	
Motamedifar *et al.* (2020)	Iran	Yes	Yes	Yes	Yes	Yes	Yes	Yes	Yes						8/8	
Pochernikov *et al.* (2020)	Russia	Yes	Yes	Yes	Yes	Yes	Yes	Yes	Yes						8/8	
Okwelogu *et al.* (2021)	Nigeria	Yes	Yes	Yes	Yes	Yes	Yes	Yes	Yes						8/8	
Yao *et al.* (2022)	China	Yes	Yes	Yes	Yes	Yes	Yes	Yes	Yes						8/8	
Garcia-Segura *et al.* (2022)	Spain	Yes	Yes	Yes	Yes	Yes	Yes	Yes	Yes						8/8	
Puerta Suárez *et al.* (2022)	Colombia	Yes	Yes	Yes	Yes	Yes	Yes	Yes	Yes						8/8	
Koort *et al.* (2023)	Estonia	Yes	Yes	Yes	Yes	Yes	Yes	Yes	Yes						8/8	
Cao *et al.* (2023)	China	Yes	Yes	Yes	Yes	Yes	Yes	Yes	Yes						8/8	
Saldarriaga López *et al.* (2024)	Colombia	Yes	Yes	Yes	Yes	Yes	Yes	Yes	Yes						8/8	
Osadchiy *et al.* (2024)	USA	Yes	Yes	Yes	Yes	Yes	Yes	Yes	Yes						8/8	
Vajpeyee *et al.* (2024)	India	Yes	Yes	Yes	Yes	Yes	Yes	Yes	Yes						8/8	
**JBI Critical Appraisal Checklist For randomized controlled trials**															**Total**	
		**Q1**	**Q2**	**Q3**	**Q4**	**Q5**	**Q6**	**Q7**	**Q8**	**Q9**	**Q10**	**Q11**	**Q12**	**Q13**	**X/13**	
Valcarce *et al.* (2017)	Spain	No	Not applicable	Yes	No	No	No	Not applicable	Yes	Not applicable	Yes	Yes	Yes	Yes	6/13	
Maretti & Cavallini (2017)	Italy	Yes	Yes	Yes	Yes	No	Yes	Yes	Yes	Yes	Yes	Yes	Yes	Yes	12/13	
Helli *et al.* (2022)	Iran	Yes	Yes	No	Yes	Yes	Yes	Yes	Yes	Yes	Yes	Yes	Yes	Yes	12/13	
Abbasi *et al.* (2021)	Iran	Yes	Yes	No	Yes	Yes	Yes	Yes	Yes	Yes	Yes	Yes	Yes	Yes	12/13	
Grande *et al.* (2022)	Italy	No	No	No	No	No	No	Yes	Yes	Not applicable	Yes	Yes	Yes	Yes	6/13	
Asadi *et al.* (2023)	Iran	Yes	Yes	Yes	Yes	No	Yes	Yes	Yes	Yes	Yes	Yes	Yes	Yes	12/13	
**QUIN tool for reviewing risk of bias *in vitro* studies**															**Total**	
		**Q1**	**Q2**	**Q3**	**Q4**	**Q5**	**Q6**	**Q7**	**Q8**	**Q9**	**Q10**	**Q11**	**Q12**		**X/24**	**%**
Barbonetti *et al.* (2011)	Italy	2	1	1	2	2	0	1	2	2	1	2	2		18	75
Barbonetti *et al.* (2013)	Italy	2	1	1	2	2	0	1	2	2	1	2	2		18	75
Raad *et al.* (2023)	Lebanon	2	1	1	2	2	0	1	2	2	1	2	2		18	75

**Table:**

JBI Critical Appraisal Checklist:	For Analytical Cross-sectional Studies	For randomized controlled trials
Q1	Were the criteria for inclusion in the sample clearly defined?	Was true randomization used for assignment of participants to treatment groups?
Q2	Were the study subjects and the setting described in detail?	Was allocation to treatment groups concealed?
Q3	Was the exposure measured in a valid and reliable way?	Were treatment groups similar at the baseline?
Q4	Were objective, standard criteria used for measurement of the condition?	Were participants blind to treatment assignment?
Q5	Were confounding factors identified?	Were those delivering treatment blind to treatment assignment?
Q6	Were strategies to deal with confounding factors stated?	Were outcomes assessors blind to treatment assignment?
Q7	Were the outcomes measured in a valid and reliable way?	Were treatment groups treated identically other than the intervention of interest?
Q8	Was appropriate statistical analysis used?	Was follow up complete and if not, were differences between groups in terms of their follow up adequately described and analyzed?
Q9		Were participants analyzed in the groups to which they were randomized?
Q10		Were outcomes measured in the same way for treatment groups?
Q11		Were outcomes measured in a reliable way?
Q12		Was appropriate statistical analysis used?
Q13		Was the trial design appropriate, and any deviations from the standard RCT design (individual randomization, parallel groups) accounted for in the conduct and analysis of the trial?

Yes / NoUnclear / Not applicable

**Table:**

	QUIN tool for reviewing risk of bias *in vitro* studies
Q1	Clearly stated aims/objectives
Q2	Detailed explanation of sample size calculation
Q3	Detailed explanation of sampling technique
Q4	Details of comparison group
Q5	Detailed explanation of methodology
Q6	Operator details
Q7	Randomization
Q8	Method of measurement of outcome
Q9	Outcome assessor details
Q10	Blinding
Q11	Statistical analysis
Q12	Presentation of results
